# 1529. Factors Associated With HIV-positive Test Results in People Who Inject Drugs

**DOI:** 10.1093/ofid/ofad500.1364

**Published:** 2023-11-27

**Authors:** Jaime Soria, Jana Collins, James R Thacker, Alice C Thornton, Ardis Hoven, Nicholas Van Sickels

**Affiliations:** University of Kentucky, Lexington, Kentucky; University of Kentucky, Lexington, Kentucky; University of Kentucky, Lexington, Kentucky; The University of Kentucky, Lexington, Kentucky; University of Kentucky, Lexington, Kentucky; University of Kentucky College of Medicine, Lexington, Kentucky

## Abstract

**Background:**

Syringe Services Programs (SSP) are essential for harm reduction among persons who inject drugs (PWID). The Kentucky Income Reinvestment Program (KIRP), a statewide program funded through the Ryan White HIV/AIDS Program, provides comprehensive early intervention services targeting those at highest risk for HIV infection. As KIRP provides testing at SSPs and at other venues serving PWID, we sought to compare factors associated with a positive HIV test result between SSP sites and community testing sites.

**Methods:**

We conducted a cross-sectional study across the state of Kentucky from January 2022 to March 2023. We compared the demographic characteristics of PWID tested across 54 SSPs versus PWID tested at KIRP outreach events (substance abuse treatment centers, correctional facilities, shelters, etc.); and factors associated with an HIV-positive test result. Study data were collected and managed using a REDCap database. The statistical analyses, including logistic regression, were performed with Stata 17 (College Station, TX: Stata Corp LLC).

**Results:**

In the study period, 20,397 PWID were tested for HIV, 10,058 in the outreach events, and 10,339 at SSPs. The median age was 39 (IQR 32 – 46) years; 11,075 (54.3%) were male, 18,905 (92.7%) were white, and 550 (6.7%) reported MSM behavior. Outreach events were superior in reaching racial and ethnic minority groups. (Table 1). The HIV-positive rate was higher in the outreach group (0.37% vs. 0.15%; p=0.003). In a multivariate model, PWID not engaged with an SSP were significantly associated with higher HIV incidence when adjusted by gender, race, and sexual risk factors for HIV acquisition.

**Table 1.**
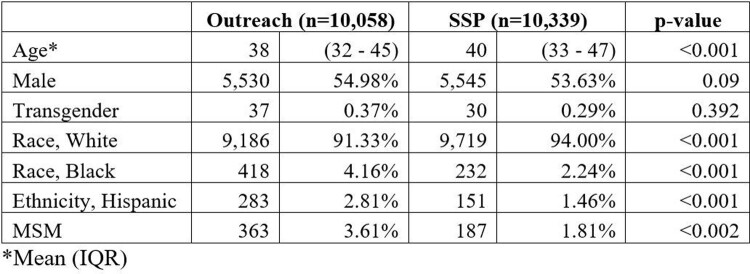
Demographic characteristics of the PWID tested for HIV by test location.

**Table 2.**
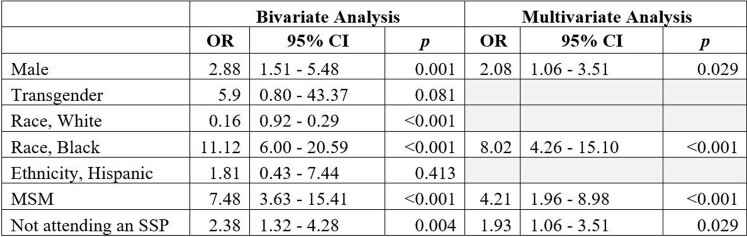
Factors associated with an HIV-positive test in PWID.

**Conclusion:**

SSPs provide comprehensive harm reduction services, and, in our study, reduced HIV incidence amongst participants. We were able to reach more underrepresented populations with testing at outreach sites, which also serve PWID. To end the HIV epidemic, and increase prevention services, enrollment of underrepresented populations into SSP services is paramount.

**Disclosures:**

**All Authors**: No reported disclosures

